# Next generation sequencing in a large cohort of patients presenting with neuromuscular disease before or at birth

**DOI:** 10.1186/s13023-015-0364-0

**Published:** 2015-11-17

**Authors:** Emily J. Todd, Kyle S. Yau, Royston Ong, Jennie Slee, George McGillivray, Christopher P. Barnett, Goknur Haliloglu, Beril Talim, Zuhal Akcoren, Ariana Kariminejad, Anita Cairns, Nigel F. Clarke, Mary-Louise Freckmann, Norma B. Romero, Denise Williams, Caroline A Sewry, Alison Colley, Monique M. Ryan, Cathy Kiraly-Borri, Padma Sivadorai, Richard J.N. Allcock, David Beeson, Susan Maxwell, Mark R. Davis, Nigel G. Laing, Gianina Ravenscroft

**Affiliations:** Harry Perkins Institute of Medical Research and the Centre for Medical Research, University of Western Australia, QQ Block, 6 Verdun Street, Nedlands, 6009, WA Australia; Genetic Services of Western Australia, King Edward Memorial Hospital, Perth, 6000, WA Australia; Victorian Clinical Genetics Services, Murdoch Children’s Research Institute, The Royal Children’s Hospital, Parkville, 3052, VIC Australia; Paediatric and Reproductive Genetics Unit, South Australia Clinical Genetics Service, Women’s and Children’s Hospital, North Adelaide, 5006, SA Australia; Department of Pediatric Neurology, Hacettepe University Children’s Hospital, Ankara, 06100 Turkey; Pediatric Pathology Unit, Hacettepe University Children’s Hospital, Ankara, 06100 Turkey; Kariminejad-Najmabadi Pathology and Genetics Centre, Tehran, 14656 Iran; Royal Children’s Hospital, Herston Road, Herson, 4029, QLD Australia; Institute for Neuroscience and Muscle Research, The Children’s Hospital at Westmead, Sydney, 2145, NSW Australia; Discipline of Paediatrics and Child Health, University of Sydney, Sydney, 2006, NSW Australia; Sydney Children’s Hospital, High Street, Randwick, 2031, NSW Australia; Unitè de Morphologie Neuromusculaire, Institut de Myologie, Institut National de la Santè et de la Recherche Mèdicale, Paris, 75651 France; Dubowitz Neuromuscular Centre, UCL Institute of Child Health, London, WC1N 1EH UK; Wolfson Centre for Neuromuscular Disorders, RJAH Orthopaedic Hospital, Oswestry, SY10 7AG UK; Department of Clinical Genetics, South Western Sydney Local Health District, Liverpool, 1871, NSW Australia; Department of Neurology, The Royal Children’s Hospital, Melbourne, 3000, VIC Australia; Genetic Services of Western Australia, Princess Margaret Hospital for Children and King Edward Memorial Hospital for Women, Subiaco, 6008, WA Australia; Department of Diagnostic Genomics, Pathwest, QEII Medical Centre, Nedlands, 6009, WA Australia; Lotterywest State Biomedical Facility Genomics and School of Pathology and Laboratory Medicine, University of Western Australia, Perth, 6000, WA Australia; Nuffield Department of Clinical Neurosciences, Weatherall Institute of Molecular Medicine, University of Oxford, Oxford, OX3 9DS UK

**Keywords:** Fetal hypokinesia, Arthrogryposis, Next generation sequencing, Congenital myopathy, Nemaline myopathy

## Abstract

**Background:**

Fetal akinesia/hypokinesia, arthrogryposis and severe congenital myopathies are heterogeneous conditions usually presenting before or at birth. Although numerous causative genes have been identified for each of these disease groups, in many cases a specific genetic diagnosis remains elusive. Due to the emergence of next generation sequencing, virtually the entire coding region of an individual’s DNA can now be analysed through “whole” exome sequencing, enabling almost all known and novel disease genes to be investigated for disorders such as these.

**Methods:**

Genomic DNA samples from 45 patients with fetal akinesia/hypokinesia, arthrogryposis or severe congenital myopathies from 38 unrelated families were subjected to next generation sequencing. Clinical features and diagnoses for each patient were supplied by referring clinicians. Genomic DNA was used for either whole exome sequencing or a custom-designed neuromuscular sub-exomic supercapture array containing 277 genes responsible for various neuromuscular diseases. Candidate disease-causing variants were investigated and confirmed using Sanger sequencing. Some of the cases within this cohort study have been published previously as separate studies.

**Results:**

A conclusive genetic diagnosis was achieved for 18 of the 38 families. Within this cohort, mutations were found in eight previously known neuromuscular disease genes (*CHRND*, *CHNRG*, *ECEL1*, *GBE1*, *MTM1*, *MYH3*, *NEB* and *RYR1*) and four novel neuromuscular disease genes were identified and have been published as separate reports (*GPR126*, *KLHL40*, *KLHL41* and *SPEG*). In addition, novel mutations were identified in *CHRND*, *KLHL40*, *NEB* and *RYR1*. Autosomal dominant, autosomal recessive, X-linked, and *de novo* modes of inheritance were observed.

**Conclusions:**

By using next generation sequencing on a cohort of 38 unrelated families with fetal akinesia/hypokinesia, arthrogryposis, or severe congenital myopathy we therefore obtained a genetic diagnosis for 47 % of families. This study highlights the power and capacity of next generation sequencing (i) to determine the aetiology of genetically heterogeneous neuromuscular diseases, (ii) to identify novel disease genes in small pedigrees or isolated cases and (iii) to refine the interplay between genetic diagnosis and clinical evaluation and management.

**Electronic supplementary material:**

The online version of this article (doi:10.1186/s13023-015-0364-0) contains supplementary material, which is available to authorized users.

## Background

### Fetal akinesia/hypokinesia

Fetal akinesia deformation sequence (FADS) or Pena Shokeir syndrome, characterized by intrauterine growth retardation, contractures, craniofacial anomalies, limb anomalies, pulmonary hypoplasia and polyhydramnios, results from reduced movement *in utero* [1, 2]. A number of other fetal akinesia syndromes overlap phenotypically with FADS. These include the lethal congenital contracture syndromes, multiple pterygium syndromes, and *arthrogryposis multiplex congenita* [3], in which the clinical findings are dependent upon the time of onset of the dyskinesia, earlier onset being associated with a more severe phenotype [2]. It’s thought that more than 50 % of all causes of fetal akinesia are of neuromuscular origin [4]; at least 30 causative genes have been identified, involving all points along the neuromuscular axis (motor neurons, peripheral nerves, neuromuscular junction and the skeletal muscle regulatory and contractile apparatus) [5–7].

### Arthrogryposis

Arthrogryposis refers to non-progressive congenital joint contractures in >1 area of the body, and has been described in more than 300 specific disorders [6, 8]. Arthrogryposis is thought to result from reduced fetal movement, and affects approximately 1 in 3,000 live births [8, 9]. There is a range of disease severity: severe cases present with *arthrogryposis multiplex congenita*, which is lethal prior to or at birth, while milder cases with a longer life expectancy may have predominantly distal involvement [8, 9]. The distal arthrogryposes are a group of disorders with contractures primarily involving the extremities of the body, often associated with camptodactyly, hypoplastic or absent flexion creases, and *talipes equinovarus* [10, 11]. There are ten distinct subtypes of distal arthrogryposis, for which seven causative genes have been identified: *ECEL1* (OMIM 605896), *MYH3* (OMIM 160720), *MYH8* (OMIM 160741), *PIEZO2* (OMIM 613692), *TNNI2* (OMIM 191043), *TNNT3* (OMIM 600692) and *TPM2* (OMIM 190990) [11–13].

### Congenital myopathies

The congenital myopathies are a diverse group of disorders, characterised by skeletal muscle dysfunction (most often weakness and hypotonia), with specific morphological features on skeletal muscle biopsies [14, 15]. Three distinct major groups are recognized based upon the presence of one or more major histopathological features: centronuclear myopathy, core myopathy and nemaline myopathy (NEM), although there is extensive overlap in both genotype and phenotype within and between these groups [16, 17]. While muscle biopsy remains critical for diagnosis, there can be overlap in the morphological abnormalities seen in these conditions, and marked variability in their clinical progression and severity [14, 15]. The clinical spectrum of the congenital myopathies ranges from severe fetal akinesia to adult-onset progressive weakness. Typical features of these conditions include proximal weakness, respiratory insufficiency, facial weakness, skeletal deformities such as hip dislocation and deformities of the feet, feeding difficulties, hypotonia and delayed motor milestones [17], however hypertonic cases are also encountered [15, 18].

More than 15 disease genes are known to cause congenital myopathies. However, many cases remain genetically unresolved, suggesting further heterogeneity [5, 7, 12, 19–21]. This study aimed to assess the potential of next generation sequencing technologies to identify causative genes in small families or isolated probands presenting with fetal hypokinesia, arthrogryposis or a severe congenital myopathy.

## Methods

### Subject information and study ethics approval

Informed consent was given for participation in this study, which was approved by the Human Research Ethics Committee of the University of Western Australia, Perth, Western Australia, Australia.

### Exome sequencing

Exome sequencing for this study was performed at the Lotterywest State Biomedical Facility Genomics Node (LSBFG) in Perth, Australia. Exome sequencing was performed on the 5500XL SOLiD™ system (Applied Biosystems), as described elsewhere [20, 22–24], and the Ion Proton™ (Ampliseq chemistry, Life Technologies) (Family 16 and 38). For AmpliSeq exome sequencing, 100 ng of DNA from the probands was amplified in 12 PCR pools and sequencing adaptors ligated. The library was then purified using AMPure beads (Beckman Coulter), and amplified using Platinum® High-Fidelity Taq Polymerase. The amplified library was again purified with AMPure beads and analysed on a 2100 Bioanalyser (Agilent Technologies Genomics). Libraries were diluted to 18-26pM and attached to Ion Sphere™ Particles using an Ion Proton™ Template 200 v3 kit and sequenced on a P1 sequencing chip on an Ion Proton sequencer™ (Ion Sequencing 200 kit v3) in pools of two.

### Targeted capture and sequencing of neuromuscular disease genes by next generation sequencing

Neuromuscular sub-exomic sequencing (NSES) was also performed at the LSBFG. The NSES panel comprised those genes listed within the December 2012 freeze of Neuromuscular Disorders gene table [25] in which the disease-causing mutations could be identified by next generation sequencing, some unpublished candidate disease genes identified by our group and others and 59 cardiomyopathy genes. NSES analysis was performed on DNA from the probands using the Ion Proton™ sequencer (Life Technologies), as previously described [26]. For NSES, 2 μg of DNA was captured in pools of 16 DNA samples using a custom TargetSeq™ (Life Technologies) capture system, enriching for the 336 known and candidate neuromuscular and cardiomyopathy disease genes. These captured pools were then sequenced in batches of 16 using an Ion P1 200 V2 sequencing kit (Life Technologies) for 520 flows.

### Bioinformatics

Variant calling was performed against the GRCh37 human reference genome, using LifeScope™ 2.5 (exome sequencing) and Torrent Suite V 3.6.2 (NSES) (Life Technologies). Data was filtered using an ANNOVAR annotation software suite. Variants were annotated using the EncodeGencode gene annotation set. Variants were filtered against the 1000 Genomes database (2012 release, [27]) and the dbSNP137 common database, and variants with a frequency of >0.5 % were excluded. Variants were then filtered against an in-house common variant list and were checked against the HGMD professional database to identify any known disease-causing mutations. The frequencies of candidate disease variants in the 1000 Genomes Project, Exome Variant Server (http://evs.gs.washington.edu/EVS/) and ExAC Browser (http://exac.broadinstitute.org) were also determined. Pathogenicity predictions were made using online prediction software programs: SIFT, PolyPhen [28], and MutationTaster [29].

The LSBFG has a cut-off of 90 % of on-target regions covered to 20-fold or greater for the neuromuscular panel (NSES) and 80 % covered to 20-fold or greater for exome sequencing, however some samples, especially early samples, did not achieve these cut-offs (Additional file 1: Table S1). There was no significant difference in the average coverage (mean ± SEM) of exome sequencing data for genetically resolved (80 ± 14-fold; *n* = *15*) versus unresolved cases (70 ± 8-fold; *n* = *23*). For the NSES panel, average coverages were 220 ± 23-fold (*n* = *6*) for resolved cases versus unresolved cases (195 ± 13-fold, *n* = *9*). Thus coverage is unlikely to contribute to the lack of a genetic diagnosis in most cases.

### Sanger confirmation and co-segregation studies

PCR amplification and Sanger sequencing was performed to verify potential mutations identified by next generation sequencing. Co-segregation was also verified for all existing family members where available. Primers were based on genomic and cDNA sequences obtained from the UCSC Human Genome Browser (http://genome.ucsc.edu/) and Ensembl (http://www.ensembl.org/). Primer sequences and conditions are available upon request. Sanger sequencing data was processed by LSBFG and results viewed using CodonCode Aligner software.

### Functional studies of the CHRND missense substitution

The mutation *CHRND* p.Cys257Arg was directly introduced into the wild-type human delta subunit cDNA in the vector pcDNA3.1/hygro (−) by site-directed mutagenesis (QuikChange® Site-Directed Mutagenesis Kit, Stratagene, Amsterdam, The Netherlands). Primer sequences can be obtained on request. To confirm the presence of the introduced mutation, and to rule out any errors, the construct was subjected to Sanger sequencing.

Wild-type and mutant human AChR δ-subunits cDNAs in the vector pcDNA3.1/hygro (−) (Life Technologies, V875–20) were used for transfection studies.

Wild-type and mutant AChR δ-subunit cDNAs, in combination with wild-type α-, β-and ɛ-subunit cDNAs, were transfected into HEK 293 cells grown on six-well tissue culture plates using polyethyleneimine. Surface AChR expression was determined 2 days post-transfection by incubating cells in 10 nM ^125^I-α-bungarotoxin (^125^I-α-BuTx) with 1 mg/ml BSA for 30 minutes. Cells were washed three times with PBS and extracted in 1 % Triton X-100, in 10 mM Tris–HCl (pH 7.4), 100 mM NaCl, 1 mM EDTA and ^125^I-α-BuTx binding determined using a gamma counter.

## Results and discussion

A total of 45 subjects from 38 families (including ten consanguineous pedigrees) diagnosed with FADS, arthrogryposis, or a severe congenital myopathy were included in this study. Of these seven probands were submitted for NSES only, eight families had probands sequenced using both NSES and exome sequencing, and 23 families underwent only exome sequencing (Additional file 1: Table S1). Families were grouped into three disease entities: FADS (*n* = *9*), arthrogryposis (*n* = *13*), and severe congenital myopathies (*n* = *16*). Clinical details for the genetically resolved families are summarized in Table 1.

**Table 1:** Summary of the clinical features of the affected individuals within each family, not described previously

ID	Consang	Case	Age at presentation	Delivery	Age (Age at death)	Birth weight (g)	Fetal akinesia/hypokinesia	Arthrogryposis	Fractures	Hydrops	Polyhydramnios	Spontaneous movements at birth	Facial movement	Orogastric Tube	Motor Milestones	Respiratory Insufficiency
16	yes	II:1	birth	C/S (35wg)	7w	1,660	yes	AMC	yes	no	yes	absent	no	yes	none	yes
20	yes	II:VI	birth	C/S (37wg)	(<1w)	NI	NI	NI	NI	NI	NI	NI	NI	NI	n/r	NI
14	no	II:2	birth	C/S	5 m	3,196	yes	no	no	no	no	hypotonic	yes	yes	delayed	yes
6	yes	II:1	birth	C/S (30wg)	(3w)	1,345	NI	AMC	no	NI	NI	absent	yes	no	n/r	yes
		II:3	32wg	C/S (36wg)	(4w)	2,345	yes	AMC	no	yes	yes	absent	yes	no	n/r	yes
8	no	II:1	25wg	*in utero* demise	(26wg)	NI	yes	AMC	no	yes	NI	n/r	yes	n/r	n/r	n/r
		II:3	29wg	*in utero* demise	(29wg)	970	yes	AMC	no	no	yes	n/r	no	n/r	n/r	n/r
13	no	II:2	birth	C/S (38wg)	5^9/12^y	3, 200	yes	no	yes	no	no	hypotonic	yes	no	delayed	no
9	yes	II:1	19 wg	TOP (19wg)	n/r	NI	NI	AMC	no	yes	NI	n/r	NI	n/r	n/r	n/r
		II:2	16wg	TOP (16wg)	n/r	98	yes	AMC	no	yes	yes	n/r	yes	n/r	n/r	n/r
		II:3	16wg	TOP (16wg)	n/r	95	yes	AMC	no	yes	yes	n/r	yes	n/r	n/r	n/r
10	no	II:1	20 wg	C/S (37wg)	(4w)	2,820	yes	AMC	no	no	yes	hypotonic	yes	yes	n/a	yes
		II:2	16wg^a^	TOP (17wg)	n/r	n/r	no	AMC	no	no	NI	n/r	NI	n/r	n/r	n/r
15	no	II:2	NI	NI	8y	NI	NI	DA	no	NI	NI	normal	no	no	normal	no
1	no	II:2	birth	C/S	4y	3504	yes	DA2A	no	no	no	normal	yes	yes	delayed	no
11	no	I:2	birth	C/S	NI	NI	no	DA2B	no	no	no	yes	no	no	NI	no
		II:1	antenatal	C/S	NI	3,410	no	DA2B	no	no	no	yes	no	no	normal	no

*C*/*S* caesaeran section, *TOP* termination of pregnancy, *wg* weeks gestation, *NI* no information, *n*/*r* not relevant, ^a^ prenatal diagnosis

A conclusive genetic diagnosis was achieved for 18/38 families (47 %, Table 2). This included two kindreds with FADS, six with arthrogryposis and 10 presenting with a congenital myopathy. From these results, autosomal dominant (*n* = *1*), autosomal recessive (*n* = *15*), *de novo* (*n* = *1*) and X-linked (*n* = *1*) modes of inheritance were identified. Mutations were identified in eight previously known neuromuscular disease genes. As part of this cohort study, four then novel disease genes were initially identified from five families (Families 3, 4, 5, 12 and 38) in the cohort and these families have been previously published: *GPR126* (Family 3) [30], *KLHL40* (Family 10 and 17; OMIM 615340) [24], *KLHL41* (Patient ID: D12-203; OMIM 607701) [22] and *SPEG* (Patient ID: P3; OMIM 615950) [20].

**Table 2:** Mutations identified via next generation sequencing

Family	Chr Position	Gene	Exon/(Intron)	Transcript	c. DNA change	Amino acid change	Alleles in ExAC
3^a,WES^	3:42728042	*KLHL40*	1	NM_152393	c.932G>T	p.Arg311Leu	Not found
	3:42730455		4		c.1516A>C	p.Thr506Pro	A:7/C:121308
4^a,WES^	3:42730521	*KLHL40*	4		c.1582G>A	p.Glu528Lys	G:8/A:120018
	3:42730521		4		c.1582G>A	p.Glu528Lys	G:8/A:120018
16^WES^	3:42727156	*KLHL40*	1		c.46C>T	p.Gln16^*^	Not found
	3:42727156		1		c.46C>T	p.Gln16^*^	Not found
20^NSES^	3:42728041	*KLHL40*	1		c.931C>A	p.Arg311Ser	C:6/A:121368
	3:42728041		1		c.931C>A	p.Arg311Ser	C:6/A:121368
5^a,WES^	2:170382132_9	*KLHL41*	6	NM_006063	c.1748_1755del8	p.Lys583Thr*fs* ^*^7	Not found
	2:170382132_9		6		c.1748_1755del8	p.Lys583Thr*fs* ^*^7	Not found
12^a,WES^	2:220331929_30	*SPEG*	12	NM_005876	c.2915_2916del2insA	p.Ala972Asp*fs* ^*^79	Not found
	2:220331929_30		37		c.8270G>T	p.Gly2757Val	Not found
38^a,WES, NSES^	6:142729324	*GPR126*	16	NM_198569	c.2306T>A	p.Val769Glu	Not found
	6:142729324		16		c.2306T>A	p.Val769Glu	Not found
14^NSES^	23:149809808	*MTM1*	8	NM_000252	c.595C>T	p.Pro199Ser	Not found
6^WES^	19:38951109	*RYR1*	20	NM_000540	c.2455C>T	p.Arg819^*^	C:1/T:121396
	19:38980890		36		c.5989G>A	p.Glu1997Lys	Not found
8^WES^	19:38987106	*RYR1*	41		c.6721C>T	p. Arg2241^*^	C:20/T:120468
	19:39071143		101		c.14645C>T	p.Thr4882Met	C:2/T:121280
13^NSES^	19:38946103	*RYR1*	15		c.1589G>A	p.Arg530His	G:8/A:121410
	19:39071143		101		c.14645C>T	p.Thr4882Met	C:2/T:121280
9^WES^	2:152539199	*NEB*	29	NM_001164508	c.2920C>T	p.Arg974^*^	Not found
	2:152539199		29		c.2920C>T	p.Arg974^*^	Not found
2^a,WES^	3:81698005	*GBE1*	(5)	NM_000158	c.691+2T>C	?	T:113/C:98680
	3:81691968		7		c.956A>G	p.His319Arg	Not found
10^WES^	2:233394798	*CHRND*	7	NM_000751	c.769T>C	p.Cys257Arg	Not found
	2:233398996		11		c.1315delG	p.Val439Trp*fs* ^*^11	Not found
15^NSES^	2:233406191_2	*CHRNG*	5	NM_005199	c.459dupA	p.Val154Ser*fs* ^*^24	CA:32/C:121406
	2:233406191_2		5		c.459dupA	p.Val154Ser*fs* ^*^24	CA:32/C:121406
1^WES^	17:10544634	*MYH3*	18	NM_002470	c.2015G>A	p.Arg672His	Not found
11^NSES^	17:10549042	*MYH3*	12		c.1123G>A	p.Glu375Lys	Not found
7^a,WES^	2:233347865	*ECEL1*	9	NM_004826	c.1531G>A	p.Gly511Ser	Not found
	2:233346560		(12)		c.1797-1G>A	?	Not found

^a^Denotes families published previously. Type of next-generation sequencing performed for each family is also noted in this table

### Mutations in fetal hypokinesia and congenital myopathy genes

#### KLHL40

**Fig. 1:**
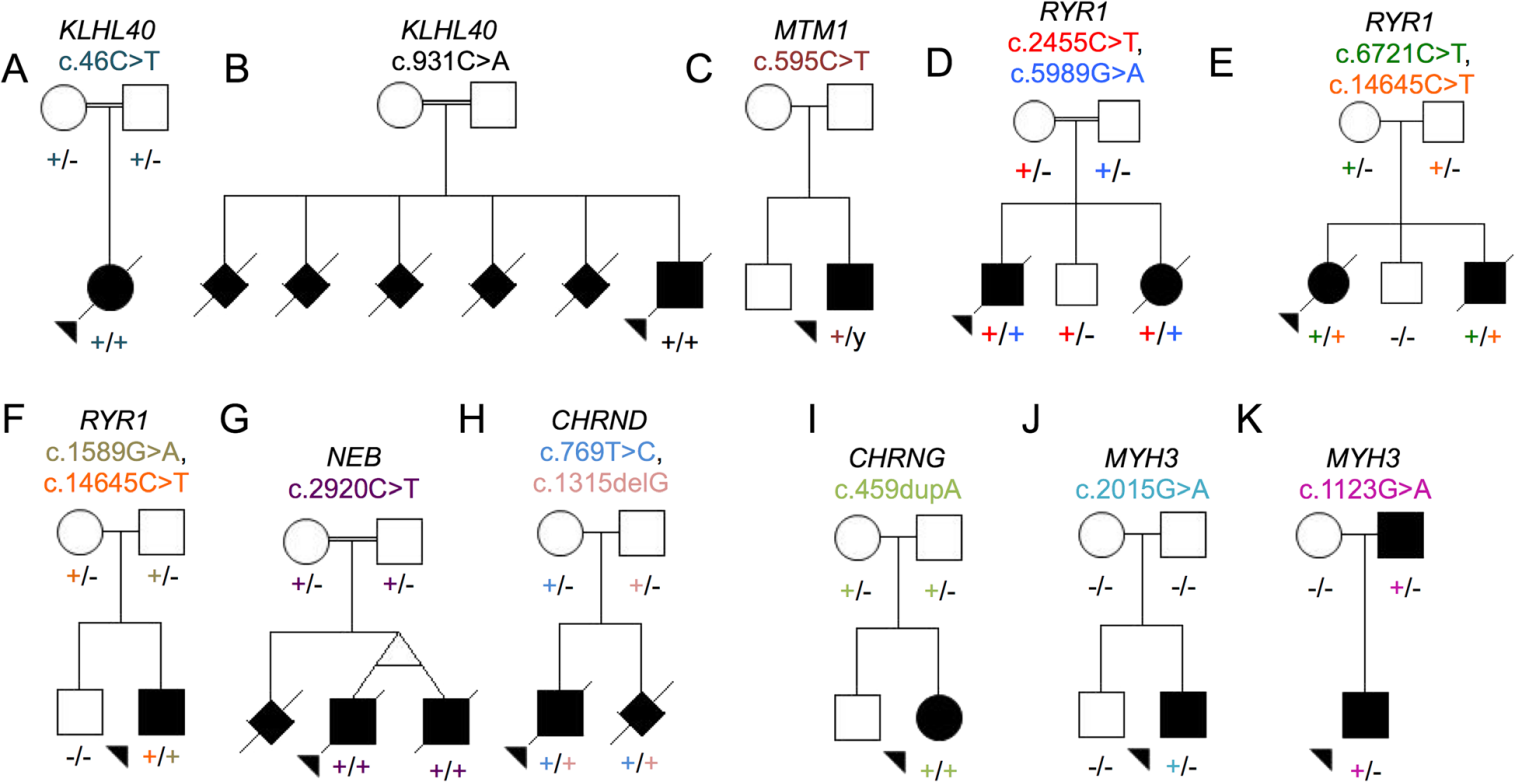
Pedigrees for families in which mutations were identified from next generation sequencing of a proband. Pedigrees and segregation of the mutation/s identified within each family is shown for pedigrees not previously described elsewhere. Probands denoted by arrowheads. (**a**) Family 16 and (**b**) Family 20 with homozygous *KLHL40* mutations; (**c**) Family 14: X-linked *MTM1* mutation; (**d**) Family 6, (**e**) Family 8 and (**f**) Family 13 with compound heterozygous mutations of *RYR1*; (**g**) Family 9: homozygous *NEB* mutation; (**h**) Family 10: compound heterozygous mutation of *CHRND*; **i** Family 15: homozygous mutation of *CHRNG*; (**j**) Family 1: *de novo* mutation of *MYH3*; (**k**) Family 11: dominantly-inherited mutation of *MYH3*. Pedigrees for Family 2^23^, 3-4^24^, 5^22^, 7^57^, 12^20^ and 38^30^ are published previously

**Fig. 2:**
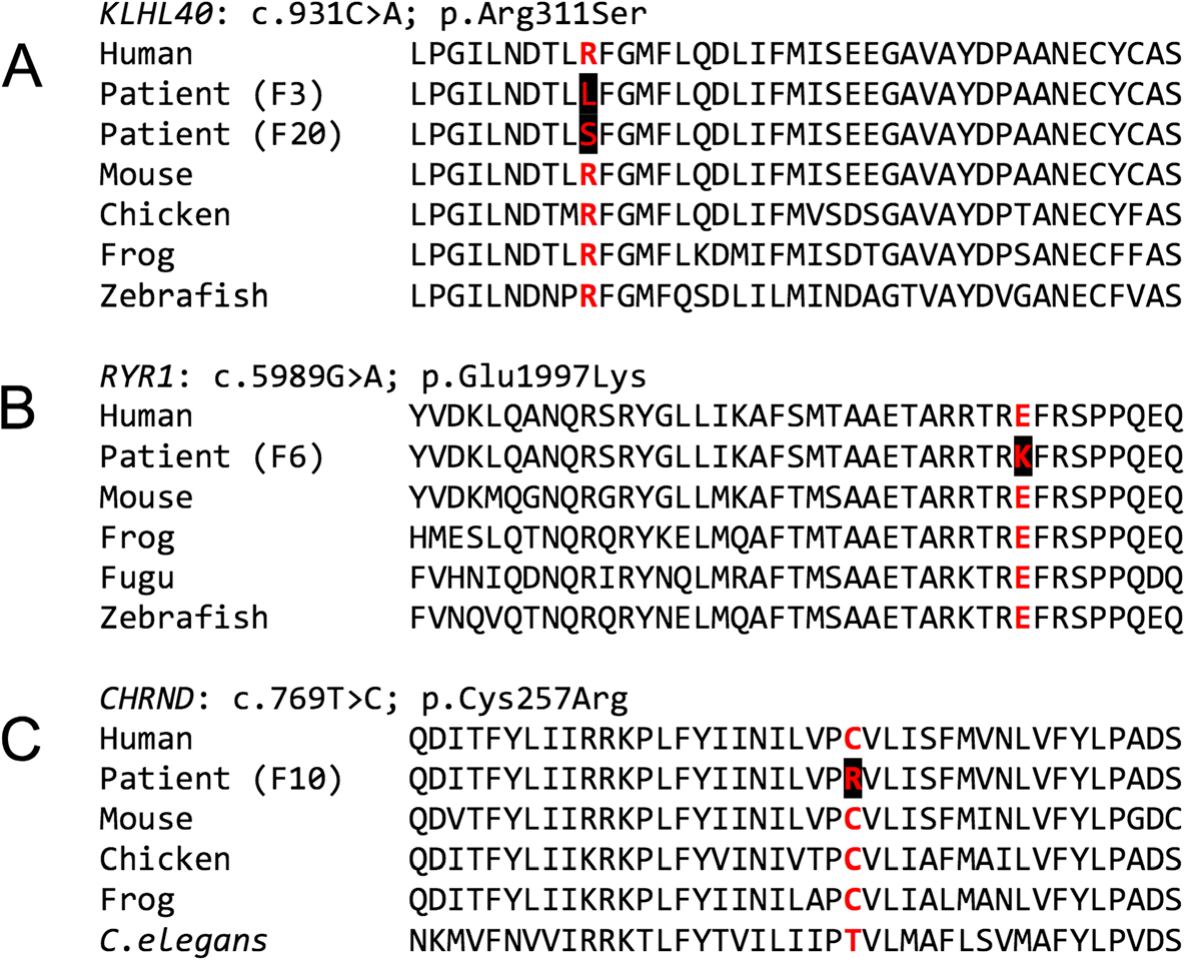
Evolutionary conservations of substituted residues in three families harbouring novel missense substitutions. Evolutionary conservation of the substituted amino acid in KLHL40 in Family 20 (**a**), RYR1 in Family 6 (**b**) and CHRND in Family 10 (**c**)

Since our initial publication of *KLHL40* as a novel NEM gene, two further families within our cohort were shown to have mutations in *KLHL40* (Families 16 and 20). A previously-unpublished homozygous nonsense mutation in *KLHL40* (exon 1, c.46C>T, p.Gln16*) was identified in a proband from consanguineous parents (Family 16, Fig. 1a). This proband was born by emergency Caesarean section at 35/40 weeks gestation and presented with severe arthrogryposis, congenital fractures, respiratory insufficiency and complete akinesia. An initial clinical diagnosis of spinal muscular atrophy type 0 was made, but both light and electron microscopy of the child’s muscle biopsy demonstrated miliary nemaline bodies (Fig. 2), adding to the body of evidence suggesting that miliary nemaline bodies are a good indicator suggesting *KLHL40* as the causative gene.

**Fig. 3:**
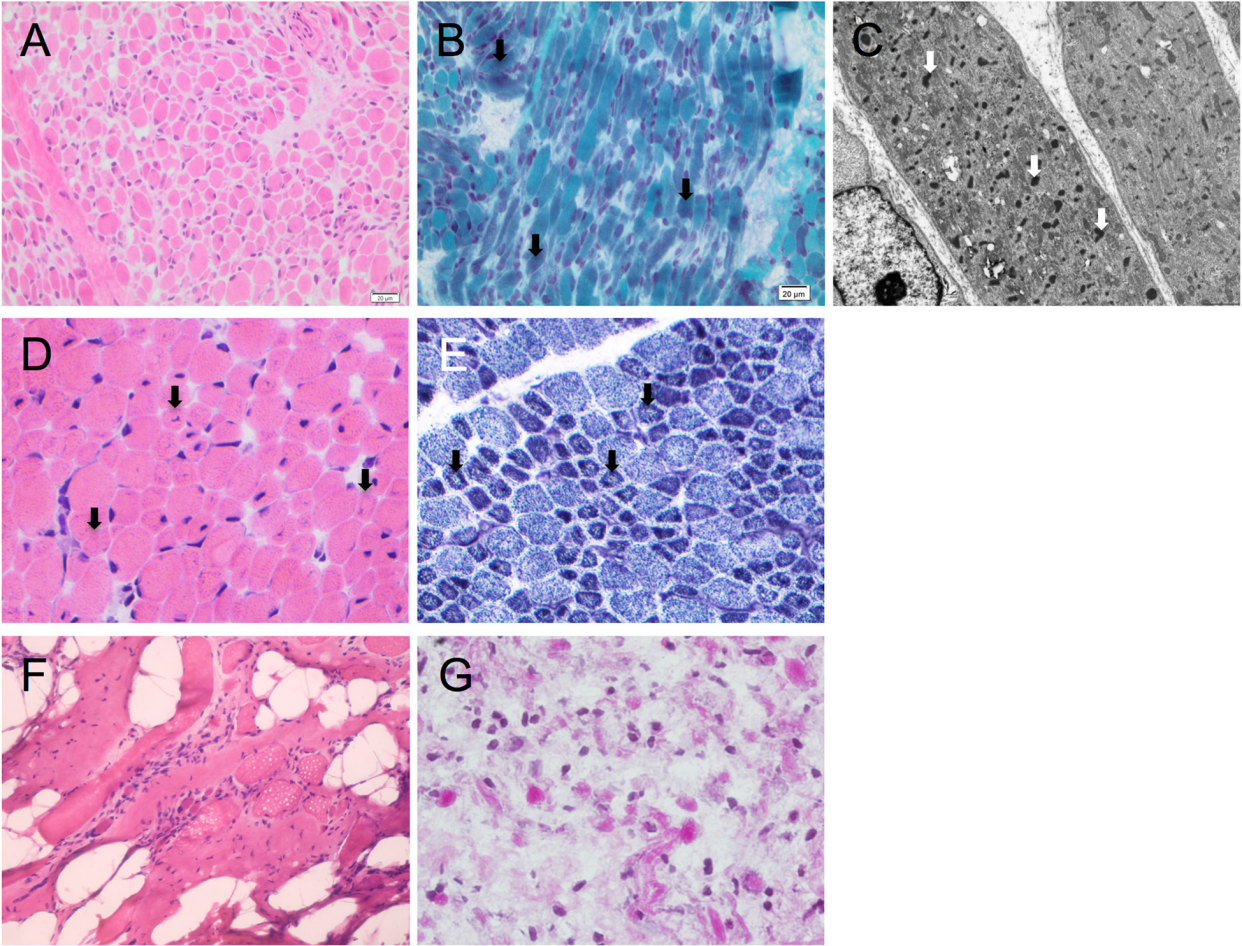
Histology of muscle biopsies from four families with mutations identified in the proband. Family 16 (**a**-**c**): **h**&**e** indicating variation in myofibre diameter (**a**) and Gomori trichrome staining showing dark purple regions suggesting nemaline bodies (arrows) (**b**). Electron micrograph, arrows indicate miliary nemaline bodies (**c**). **(d)** H&E stain of muscle from the proband in Family 14, indicating variation in myofibre size, central and internal nuclei. **(e)** Staining for NADH-TR in muscle from the proband in Family 14 with arrows indicating reduced central staining indicative of minicores. **(f)** H&E staining of muscle from the proband in Family 13 showing muscle tissue embedded in fibro-adipose tissue, with severe myopathic, non-specific changes. **(g)** H&E staining of muscle from the proband in Family 8, demonstrating a severe non-specific picture

The proband in Family 20 was born to consanguineous parents (Fig. 1b) by Caesarean section at 37 weeks gestation. He had profound hypotonia, an absent gag reflex, myopathic facies, and was ventilated from birth, but survived only a few days. His muscle biopsy showed numerous nemaline bodies. The family history included two previous miscarriages, two neonatal deaths and a sibling who died at seven months of age with suspected NEM (light microscopy indicated rods, but electron microscopy was not performed). No mutations were found on Sanger sequencing of *ACTA1*, but NSES showed a novel homozygous missense mutation in *KLHL40* (exon 1, c.931C>A, p.Arg311Ser) affecting the same highly conserved amino acid residue as that in Family 3 (Fig. 3a).

#### MTM1

The second male child of a non-consanguineous family (Family 14, Fig. 1c) was born after an uncomplicated pregnancy, by emergency Caesarean section for failure to progress. The baby was weak and hypotonic at birth, was very long (reported >90th percentile for length with weight 10-25th percentile), had advanced bone age, and initially required intubation. By age 5 months the infant’s strength and spontaneous movement improved markedly, but he had significant residual weakness and bulbar dysfunction. NSES identified a known missense mutation (exon 8, c.595C>T, p.Pro199Ser) in the myotubularin gene (*MTM1*; OMIM 300415) [31] associated with myotubular myopathy. A muscle biopsy taken at 10 weeks of age revealed hypoplastic myofibres, some with internal nuclei, typical features of myotubular myopathy (OMIM 310400) [32]. However, enzyme staining showed reduced central staining in some myofibres, while electron microscopy showed foci of sarcomeric dissolution, suggestive of cores. IHC for myosin confirmed the preservation of type II/fast myofibres and numerous small type I myofibres. Thus a diagnosis of congenital myopathy with fibre-type disproportion and occasional minicores had been suggested (Fig. 2d-e). This highlights that *MTM1* cases can present with congenital weakness and muscle biopsies displaying features of fibre type disproportion and minicores.

#### RYR1

The proband and affected sibling of Family 6 (Fig. 1d), were born to consanguineous parents. The proband was born at 30 weeks gestation with profound hypotonia, facial weakness, dysmorphic features and ambiguous genitalia, after a pregnancy complicated by fetal hypokinesia. He died at 3 weeks of age. A subsequent pregnancy with a female sibling was complicated by polyhydramnios. At birth there was minimal limb movement, respiratory distress necessitating mechanical ventilation, subcutaneous oedema, contractures of the hips and knees and camptodactyly of the fingers. She died at 4 weeks of age. Maternal testing for myotonic dystrophy (*DM1*) was negative. Vastus lateralis biopsies from both babies showed non-specific abnormalities of myofibre typing, with type II myofibre predominance and numerous small myofibres. Occasional minicores and cores were seen in the proband but not his sibling. Neither had nemaline bodies or histologic features of myotubular myopathy. Exome sequencing performed on the proband revealed two mutations in the ryanodine receptor gene (*RYR1*, OMIM 180901): a novel heterozygous missense mutation affecting a highly conserved amino acid (Fig. 3b) (exon 36, c.5989G>A, p.Glu1997Lys) and a heterozygous previously-reported nonsense mutation (exon 20, c.2455C>T, p.Arg819* [33]). Sanger sequencing confirmed these mutations and showed co-segregation with disease. Thus in this instance, the consanguinity does not appear to be a contributing factor in the siblings’ disease. The nonsense mutation was previously identified in a 49-year old ambulant patient with a moderate form of slowly-progressive myopathy with cores [33]. That patient also harboured a previously identified heterozygous missense mutation (p.Arg4558Gln) [33, 34]. Thus the same nonsense mutation, in combination with different missense mutations, can result in variable phenotypes, from fetal hypokinesia and death in the perinatal period, to a mild delay in motor milestones and normal life expectancy.

The proband in non-consanguineous Family 8 (Fig. 1e) presented with non-immune hydrops fetalis and arthrogryposis, and was stillborn at 26 weeks gestation. Autopsy showed multiple contractures and reduced muscle bulk. Microscopically, there were marked dystrophic changes in all muscles examined (Fig. 2g). The contactin-1 gene (*CNTN1*, OMIM 600016) was Sanger sequenced but no mutations were identified. A subsequent pregnancy with a male fetus was complicated by polyhyhydramnios, contractures, and *in utero* fetal demise at 29 weeks gestation. Both affected individuals were diagnosed with FADS and congenital muscular dystrophy. Exome sequencing of the proband identified two previously reported heterozygous mutations in the *RYR1* gene; a nonsense mutation (exon 41, c.6721C>T, p.Arg2241* [35]) and a missense mutation (exon 101, c.14645C>T, p.Thr4882Met [36]) associated with multiminicore disease and core rod disease, respectively. Sanger sequencing confirmed compound heterozygosity in both affected individuals, and showed that both parents were carriers and that the unaffected sibling did not harbour either mutation.

The affected individual in Family 13 was born to non-consanguineous Turkish parents (Fig. 1f) after reports of reduced intrauterine movement. He was delivered at term by Caesarean section, due to poor positioning. At birth bilateral humeral fractures were noted. He had a diagnosis of *osteogenesis imperfecta*, and followed up with alendronate treatment. He was referred to pediatric neurology outpatient clinic at the age of 19 months when the parents had concerns in terms of hypotonia and delay in motor developmental milestones. At the time, he remained hypotonic with a myopathic face and high-arched palate. He had axial and vertical hypotonia, head lag, facial weakness, and absence of deep tendon reflexes. He could sit but not stand. The muscle biopsy showed muscle tissue embedded in fibro-adipose tissue with severe non-specific myopathic changes (Fig. 2f). There were hypertrophic and atrophic myofibres, central nuclei, type II myofibre predominance and some core-like regions on oxidative enzyme stains. Exome sequencing of the proband revealed two pathogenic missense mutations in the *RYR1* gene, (exon 15, c.1589G>A, p.Arg530His [37], exon 101, c.14645C>T, p.Thr4882Met [36]) which had previously been associated with central core disease/malignant hyperthermia (MH) and core rod myopathy, respectively. The p.Arg530His substitution was inherited paternally, thus the presence of this MH (OMIM 145600) susceptibility mutation in both the proband and asymptomatic father changes their clinical management.

Thus, affected individuals in three families (Family 6, 8, and 13), harbored compound heterozygous mutations in *RYR1*. Disease severity was much greater in the two families possessing a nonsense (null) mutation as well as a missense mutation (Family 6 and 8), resulting in death at or soon after birth. The affected individual in the third *RYR1* family, (Family 13), possessed two missense mutations, and survived infancy, albeit with severe muscle weakness and motor delay. He had a rather static improving course with physiotherapy. These findings mirror those of recent publications expanding the phenotypes associated with recessive *RYR1* disease to include *arthrogryposis multiplex congenita* and fetal akinesia [9, 38, 39]. Despite *RYR1* originally being described as a disease gene for central core disease and minicore disease, cores are seen in only a minority of recessive *RYR1* cases, and are less likely to be seen in cases with hypomorphic (null) muations [38, 39]. In this study, cores were not a prominent feature in two of the recessive *RYR1* families, both of which harboured a hypomorphic mutation.

#### NEB

A consanguineous family (Family 9) presented early in pregnancy with monoamniotic male twins (Fig. 1g) and a history of a previous fetus therapeutically aborted due to hydrops fetalis at 19 weeks gestation. Ultrasound scanning revealed severe hydrops in both fetuses, and the pregnancy was terminated at 16 weeks gestation. Post-mortem analysis of both twins showed bilateral joint contractures, bilateral talipes, multiple pterygia, hypertelorism and cystic hygromas. Muscle biopsies were not taken. A diagnosis of fetal akinesia with lethal multiple pterygia syndrome was made. Karyotyping showed a normal 46XY karyotype, with no apparent genomic imbalance. Exome sequencing was performed on one twin, and a novel homozygous nonsense mutation (exon 29, c.2920C>T, p.Arg974*) in the nebulin gene (*NEB*; OMIM 161650) was identified. Sanger sequencing confirmed that both twins were homozygous for this mutation and that each parent was a carrier (Fig. 1). This mutation was included in the recent *NEB* mutation update [40]. Although this case was diagnosed as FADS/lethal multiple pterygia syndrome, recessive mutations in the *NEB* gene are a known cause of NEM, which in severe cases can have a FADS phenotype [41]. Without a muscle biopsy however, it cannot be determined whether these cases had nemaline myopathy.

In three additional families, diagnosed with NEM presenting with fetal akinesia, single heterozygous pathogenic mutations were identified in *NEB* by either exome sequencing or NSES (Table 3). In Family 17 a known splice-site mutation (intron 5, c.78+1G > A, [42]) was identified, and in Family 19 a known frameshift mutation (exon 55, c.7523_7526del4, p.Ile2508Thrfs*14, [43]), was identified, both of which are associated with NEM. In Family 18, a previously unpublished nonsense mutation (exon 29, c.2864G>A, p.Trp955*) was identified. A common deletion of exon 55 of *NEB*, originating within the Ashkenazi Jewish population, is known to cause a severe NEM phenotype [44]. A heterozygous deletion of this exon would not be identifiable through next generation sequencing techniques. Deletion analysis was performed on the affected individuals of Family 17 and Family 18, which confirmed they did not have a deletion of this exon. The proband in Family 19 could not have harboured a deletion of exon 55, since the exon 55 variant identified in this proband was heterozygous. Although only single heterozygous mutations were identified in these three severe NEM cases, given their severity and the absence of likely pathogenic variants in the other known NEM genes, it is likely that they are harbouring a second pathogenic *NEB* variant that was not identified by next generation sequencing. In support of AR NEM, Family 18 and 19 both had a previously affected fetus. In further support that these cases (three of nine NEM families, 33 %) are harbouring an additional pathogenic *NEB* variant, only one truncating *NEB* variant was identified by next generation sequencing in non-NEM cases, of which we have sequenced and analysed in excess of >500 probands (~0.2 %). Due to the highly repetitive nature of exons 83–105 of *NEB*, next generation sequencing is unable to accurately sequence and map this region; in addition, next generation sequencing data is not reliable for the detection of small CNVs. However a targeted *NEB* array CGH has been developed as an adjunct to overcome these limitations [45] and has recently identified a recurrent CNV within this triplicated repeat [46].

**Table 3:** Single heterozygous mutations identified in NEB in three families presenting with fetal hypokinesia-NEM

Family	Exon/(Intron)	c. DNA change NM_001164507.1	Amino acid change	Comments
17	(5)	c.78+1G>A		
18	29	c.2864G>A	p.Trp955^*^	Also present in affected sib
19	55	c.7523_7526del4	p.Ile2508Thr*fs* ^*^	Affected sib (no DNA available)

Therefore, of the nine NEM cases in our cohort, five cases had mutations in the newly described genes *KLHL40* and *KLHL41*, and an additional three cases are thought likely to harbour a second pathogenic mutation in *NEB*. It is likely that many undiagnosed NEM cases are due to mutations in *NEB*, however due to its size it has not been routinely screened. With the introduction of next generation sequencing techniques, more *NEB*-related NEM cases are beginning to be identified. This may mean that there are not as many new NEM genes to find as might have been thought.

#### GBE1

A non-consanguineous family (Family 2) presented with recurrent fetal akinesia and multiple pterygium syndrome [23]. We identified compound heterozygous mutations in the gene *GBE1*, a known splice site mutation (intron 5, c.691+2T>C) associated with a non-lethal neonatal glycogenosis type IV, and a missense mutation (exon 7, c.956A>G, p.His319Arg). This report extended the phenotypic spectrum of *GBE1* disease to include lethal multiple pterygium syndrome [23].

### Mutations in known disease genes for arthrogryposes

#### CHRND

The proband in Family 10 was the first child to non-consanguineous parents, born following an IVF pregnancy, (Fig. 1h). A routine 20-week ultrasound identified bilateral fetal talipes. Chromosome microarray was normal. The fetal phenotype evolved with polyhydramnios, fetal micrognathia and an absence of hand movements noted at 32 weeks. The polyhydramnios required three amnioreduction procedures. The male infant was delivered by elective Caesarian section for placenta praevia at 37 weeks gestation and weighed 2.82 kg. He was intubated and ventilated at 10 minutes for apnoea and poor respiratory effort after APGARS of 5^1^, 6^5^ and 7^10^. He had micrognathia, cryptorchidism, a left single palmar crease, bilateral talipes, moderate large joint contractions, hypotonia, an absent gag/suck, and paucity of movement. He developed a weak suck and infrequent antigravity movement of the fingers after a week. Prader-Willi syndrome, SMA and myotonic dystrophy were excluded. Endocrine and metabolic investigations were normal as was the ophthalmologic examination. Brain MRI showed a right MCA infarct in the context of positive maternal serology for SLE. Multiple attempts to extubate the patient to CPAP failed. Ongoing ventilatory support was considered futile and was withdrawn at 4 weeks of age. Exome sequencing was performed and two novel heterozygous mutations were identified in *CHRND* (OMIM 100720) that encodes the delta-subunit of the acetylcholine receptor (AChR) [47]. A missense mutation (c.769T>C) in exon 7 that resulted in substitution of a highly conserved amino acid (p.Cys257Arg, Fig. 3c) and a frameshift mutation in exon 11 (c.1315delG, p.Val439Trpfs*11). To our knowledge neither of these mutations have been previously reported and are not listed in the *CHRND* locus-specific database (http://www.dmd.nl/nmdb/home.php?select_db=CHRND). Sanger sequencing confirmed the presence of the mutations in the affected individual and showed each parent was a carrier of one of the variants. The mother conceived a second time, naturally. Prenatal diagnosis was performed and the fetus had both variants. The pregnancy was terminated.

**Fig. 4:**
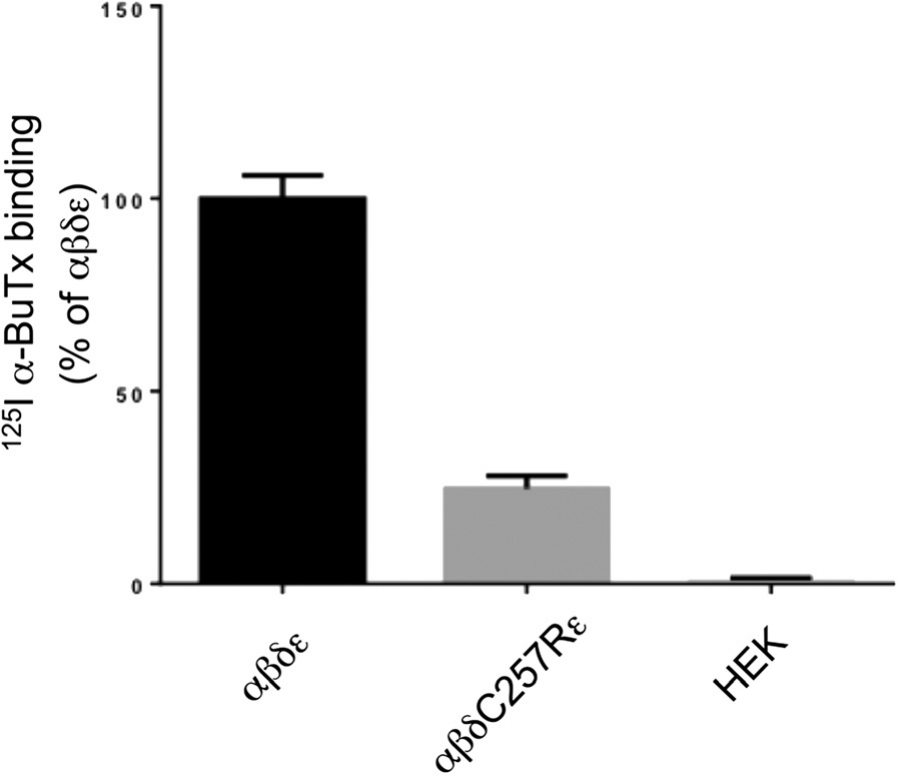
Expression of wild-type (αβδε) and mutant (αβδC257Rε) acetylcholine receptors (AChR) in HEK 293 cells. AChR expression was determined through the binding of ^125^I α-Bungarotoxin (^125^I α-BuTx) to AChR on the cell surface (*n* = 6). Note: numbering of the mutation includes the pre-peptide sequence

Studies in HEK cells, found that cell surface expression levels of AChRs harbouring the δC257R subunit to be approximately 20 % of wild-type (Fig. 4). This result is consistent with the c.769T>C mutation (in combination with c.1315delG, p.Val439Trpfs*11 on the second allele) underlying a congenital myasthenic syndrome due to AChR deficienc*y*. The mother is currently pregnant and is approaching term with a healthy fetus following PGD. Mutations of *CHNRD* typically result in congenital myasthenic syndromes (OMIM 608930 (fast-channel) and 601462 (slow-channel) [48, 49]). but have also more recently been associated with lethal multiple pterygium syndrome [50]. In two families presenting with recurrent lethal multiple pterygium syndrome, resulting in terminations during the second trimester of pregnancy, null mutations of *CHRND* were identified (one consanguineous family with a homozygous p.Trp57* mutation and one with compound heterozygous p. Phe74Leu and p.Arg464* mutations). Substitutions of amino acids in close proximity to Cys257 have been shown to cause congenital myasthenia and impaired channel function (p.Pro250Gln [51] and p.Ser268Phe [48]).

#### CHRNG

The affected female individual in Family 15 was born to unrelated parents (Fig. 1i). At birth there was arthrogryposis with distinctive shin dimples. The clinical picture of this patient is presented in Hall et al., (Patient 10) [52]. NSES was performed on the proband and revealed a known frequent homozygous frameshift mutation (exon 5, c.459dupA, p. Val154Serfs*24) in the gene encoding the gamma-subunit of the AChR (*CHRNG*; OMIM 100730) [53, 54]. Sanger sequencing confirmed the presence of the mutation in the affected individual, as well as showing each parent had the mutation in the heterozygous state. Given the unique presentation of arthrogryposis with shin dimples in this case and others harbouring *CHRNG* mutations [54], *CHRNG* should be considered in individuals presenting with this particular phenotype.

#### MYH3

The proband in Family 1 was born from unaffected parents and has an unaffected sibling (Fig. 1j). He presented with Freeman-Sheldon Syndrome (DA2A, OMIM 193700) [55] and on examination at 2 years of age he showed some facial features and proximal weakness. Exome sequencing of the proband demonstrated heterozygosity for a mutation in *MYH3* (exon 18, c.2015G>A, p.Arg672His; OMIM 160720 [55];) previously associated with Freeman-Sheldon syndrome. Sanger sequencing confirmed the presence of this mutation in the proband as well as its absence in the unaffected sibling and both unaffected parents, confirming the mutation was *de novo*.

The male proband from Family 11, was born from an unaffected mother, but affected father (Fig. 1k). Both proband and the father were born with a very typical Sheldon-Hall distal arthrogryposis (DA2B) phenotype. Sheldon-Hall syndrome can be caused by autosomal dominant or *de novo* mutations in a number of genes. In this kindred, screening of *TPM2* and *TNNI2* identified no mutations. On NSES, however, a known heterozygous *MYH3* mutation (exon 12, c.1123G>A, p.Glu375Lys [55]) previously associated with Freeman-Sheldon syndrome, was identified. Sanger sequencing confirmed the mutation in both the proband and his affected father, confirming autosomal dominant inheritance.

#### ECEL1

Non-consanguineous Family 7, previously described in [56], was also part of this cohort study. The proband was born from an uncomplicated pregnancy with extended hips, multiple arthrogrypotic features, multiple pterygium, adducted thumbs and bilateral ptosis. The couple presented when pregnant again, and on ultrasound at 20 weeks the fetus appeared to have similar features to those of the proband. The pterygia and ptosis led to consideration of multiple pterygium syndrome (Table 1). Exome sequencing revealed compound heterozygous mutations in *ECEL1* (OMIM 605896), a missense substitution (c.1531G>A, p.Gly511Ser) and a essential splice-site mutation (c.1797-1G>A). Mutations in *ECEL1* are associated with distal arthrogryposis type 5D (OMIM 615065), and the clinical presentation was in keeping with those recently described for DA5D [13, 57], although pterygia was a more prominent feature in this family.

In another cohort study, Laquerriere et al. identified two novel genes (*CNTNAP1* and *ADCY6*) for severe *arthrogryposis multiplex congenita* (AMC) by exome sequencing, and achieved a genetic diagnosis for 24 of 31 multiplex and/or consanguineous AMC families studied (>75 %). This highlights the importance of working with well-phenotyped cohorts [9]. Mutations in *CNTNAP1* were identified in four of their 31 families, suggesting that mutations in this gene underlie a significant proportion of recessive AMC cases.

Results from our study, and that of Laquerriere et al., suggest that there are further arthrogryposis disease genes to be identified [9]. ADCY6 and CNTNAP1 are both involved in axonal function [9], as is ECEL1 [57]. GPR126 is critical for myelination of peripheral nerves [58] and we identified AMC patients with loss-of-function mutations in *GPR126* [30]. Genes involved in axonal function should therefore be considered as candidates for arthrogryposis, in addition to skeletal muscle contractile proteins.

Our study highlights the widening spectrum of phenotypes associated with mutations in known fetal akinesia, arthrogryposis and myopathy genes, as is increasingly demonstrated for other neuromuscular disorders [26, 59, 60]. As sequencing of targeted gene panels or exome sequencing becomes the mainstay of genetic diagnostics [61, 62], it is likely that there will be greater broadening of genotype-phenotype correlations for neuromuscular diseases. With the overwhelming amount of genetic information obtained via next generation sequencing, the reliability of meticulously curated locus-specific databases, the availability of large exome datasets from ethnically matched reference populations and appropriate functional and/or protein studies will be critical to obtaining accurate genetic diagnosis. Given that numerous novel disease genes and mutations are being described in non-Caucasian inbred populations [63] and genetic isolates [64], there is a real need for exome sequencing of healthy individuals within these populations.

Within our cohort, three novel disease genes were initially identified by exome sequencing of single probands (*GPR126*, *KLHL41* and *SPEG*). The success of disease gene discovery in NEM (*KLHL40*, *KLHL41*, *LMOD3*, *MYO18B*) and centronuclear myopathies (*SPEG*) is likely due to the ability to identify patients with a very similar presentation (clinically and based upon very specific muscle biopsy findings) such as to enable screening of candidate genes in patients with the same disease [21, 65].

For fetal hypokinesia and arthrogryposis cases, it is more difficult to deeply phenotype the patients, due in many cases to the poor preservation of fetal tissue and the lack of specific pathological hallmarks from biopsy or autopsy material. A recent study describes exome sequencing of 143 multiplex consanguineous families, in which 33 novel candidate neurogenic disease genes were identified [63], highlighting the value of studying consanguineous families. As a comparison, only three of the 20 (15 %) genetically-unresolved cases were consanguineous whereas seven of 18 of the genetically-diagnosed cases were consanguineous (39 %, Additional file 1: Table S1), thus one is 2.5-times more likely to identify the causative disease gene in consanguineous families. A genetic diagnosis was achieved in ten of 16 congenital myopathy cases (63 %) and six of 13 arthrogryposis cases (46 %) but only 22 % of fetal akinesia cases (two of nine). It is also possible that the cause of disease, in some of the isolated cases (particularly those diagnosed with fetal akinesia), is not due to a monogenic disorder but may be environmental and/or polygenic. In families with multiple affected siblings and normal CGH arrays, we will pursue whole genome sequencing and/or RNA-seq of target tissue cDNA to try to identify novel disease genes and/or mechanisms.

## Conclusions

In summary, this study highlights the use of next generation sequencing to genetically diagnose 47 % of cases within a heterogeneous severe neuromuscular disease cohort. The study has also resulted in the identification of four novel neuromuscular disease genes, and has led to the identification of a novel mechanism of sarcomere assembly and muscle dysfunction involving *KLHL40*, *KLHL41* and *LMOD3* [21, 66, 67]. Finally, this study has contributed to extending the phenotypic spectrum of *CHRNG*, *ECEL1*, *GBE1* and *RYR1*.

## Additional file

Additional file 1: Table S1. For each family the affected individuals sequenced are shown as well as the type of NGS that was performed for each patient. The disease group and consanguinity for each family is also indicated as are the coverage statistics for each sample and NGS. (DOCX 21 kb)

## Abbreviations

AMC: Arthrogryposis multiplex congenita
DA: Distal arthrogryposis
FADS: Fetal akinesia deformation sequence
LSBFG: Lotterywest state biomedical facility genomics node
NEM: Nemaline myopathy
NSES: Neuromuscular sub-exomic sequencing
