# Supplementary material for: Next generation sequencing in a large cohort of patients presenting with neuromuscular disease before or at birth
Source: Orphanet J Rare Dis. 2015 Nov 17;10:148. doi: 10.1186/s13023-015-0364-0 (PMC4650299; doi:10.1186/s13023-015-0364-0)
Supplement: Additional file 1: Table S1. — For each family the affected individuals sequenced are shown as well as the type of NGS that was performed for each patient. The disease group and consanguinity for each family is also indicated as are the coverage statistics for each sample and NGS. (DOCX 21 kb) [file 13023_2015_364_MOESM1_ESM.docx]

| **Additional file 1: Table S1**: For each family the affected individuals sequenced are shown as well as the type of NGS that was performed for each patient. The disease group and consanguinity for each family is also indicated as are the coverage statistics for each sample and NGS. | | | | | | |
| --- | --- | --- | --- | --- | --- | --- |
| ID | D-# | NSES (average coverage, % covered to >20x) | WES_SOLID (average coverage, % covered to >20x) | WES_AmpliSeq (average coverage, % covered to >20x) | Consang | Disease |
| **UNRESOLVED** | | | | | | |
| 17 | D12-531 | 219-fold, 93% | 29-fold, 57% |  |  | CM-NEM |
| 18 | D01-275 |  | 61-fold, 80% |  |  | CM-NEM |
| 19 | D13-895 | 181-fold, 91% |  |  |  | CM-NEM |
| 21 | D13-564 | 198-fold, 93% |  | 54-fold, 83% |  | CM-NEM |
| 22 | D13-598 | 217-fold, 91% |  | 132-fold, 93% |  | CM-NEM |
| 23 | D12-014 |  | 48-fold, 69% |  |  | CM |
| 24 | D12-329 | 143-fold, 82% | 47-fold, 77% |  | Yes | FADS |
|  | D12-330 |  | 76-fold, 90% |  |  |  |
| 25 | D12-950 |  | 49-fold. 78% |  |  | FADS |
|  | D12-953 |  | 61-fold, 86% |  |  |  |
| 26 | D12-403 |  | 85-fold, 82% |  |  | FADS |
|  | D12-434 |  | 99-fold, 78% |  |  |  |
| 27 | D12-1012 | 265-fold, 93% | 54-fold, 86% |  |  | FADS |
| 28 | D12-549 |  | 54-fold, 86% |  | Yes | FADS |
| 29 | D13-090 |  | 65-fold, 88% |  | Yes | FADS |
| 30 | D12-558 |  | 53-fold, 86% |  |  | FADS |
| 31 | D12-928 | 147-fold, 91% | 219-fold, 95% |  |  | Arthrogryposis |
| 32 | D11-003/ D14-0067 | 176-fold, 93% | 49-fold, 77% |  |  | Arthrogryposis |
| 33 | D11-487 |  | 65-fold, 73% |  |  | Arthrogryposis |
| 34 | D12-1022 |  | 61-fold, 81% |  |  | Arthrogryposis |
|  | D12-1023 |  | 59-fold, 78% |  |  |  |
| 35 | D12-999 | 212-fold, 92% |  |  |  | Arthrogryposis |
| 36 | D13-091 |  | 57-fold, 87% |  |  | Arthrogryposis |
| 37 | D12-498 |  | 51-fold, 81% |  |  | Arthrogryposis |
|  | D12-500 |  | 73-fold, 87% |  |  |  |
| **RESOLVED** | | | | | | |
| 3 | D09-921 |  | 97-fold, 86% |  |  | CM-NEM |
| 4 | D10-450 |  | 72-fold, 76% |  | Yes | CM-NEM |
|  | D10-453 |  | 61-fold, 74% |  |  |  |
| 16 | D13-1010 |  |  | 40-fold, 56% | Yes | CM-NEM |
| 20 | D13-1552 | 282-fold, 95% |  |  | Yes | CM-NEM |
| 5 | D12-203 |  | 53-fold, 64% |  | Yes | CM-NEM |
| 12 | D12-539 |  | 52-fold, 86% |  |  | CM |
| 38 | D13-546 | 223-fold, 93% |  | 131-fold, 93% | Yes | Arthrogryposis |
| 14 | D13-660 | 230-fold, 92% |  |  |  | CM |
| 6 | D12-487 |  | 50-fold, 75% |  | Yes | CM |
| 8 | D12-753 |  | 93-fold, 78% |  |  | CM |
| 13 | D12-542 | 256-fold, 94% |  |  |  | CM |
| 9 | D12-060 |  | 255-fold, 96% |  | Yes | FADS |
| 2 | D11-485 |  | 67-fold, 75% |  |  | FADS |
|  | D11-486 |  | 85-fold, 79% |  |  |  |
| 10 | D12-1045 |  | 59-fold, 85% |  |  | Arthrogryposis |
| 15 | D13-1032 | 125-fold, 89% |  |  |  | Arthrogryposis |
| 1 | D12-831 |  | 40-fold, 72% |  |  | Arthrogryposis |
| 11 | D12-998 | 205-fold, 92% |  |  |  | Arthrogryposis |
| 7 | D12-706 |  | 45-fold, 74% |  |  | Arthrogryposis |
